# Transesophageal Echocardiographic Diagnosis and Management of Circumflex Artery Injury Following Mitral Valve Repair

**DOI:** 10.4021/cr22e

**Published:** 2011-03-25

**Authors:** Chinnamuthu Murugesan, Bheemaiah Raghu, Parachuri Venkateshwara Rao

**Affiliations:** a Department of Anesthesiology and Cardiac Surgery, Narayana Hrudayalaya Hospitals, No. 258/A, Bommasandra industrial area, Anekal Taluk, Bangalore-560099, India

**Keywords:** Mitral valve repair, Mitral regurgitation, Transesophageal echocardiography

## Abstract

A 16-year-old woman with severe mitral regurgitation as a result of rheumatic heart disease underwent mitral valve repair with posterior mitral annuloplasty. ST elevation was observed in leads II, III and aVF after weaning from cardiopulmonary bypass. On transesophageal echocardiography, the stenosis of the circumflex artery was suggested by a modified midesophageal long axis. Since the patient was hemodynamically unstable, an emergency coronary angiography could not be considered. An urgent cardiopulmonary bypass was re-instituted; the first two sutures in the P1 region of the posterior mitral annulus were translocated more superficially. Transesophageal echocardiography revealed good opening of the circumflex artery and improvement in regional wall motion abnormality following the corrected procedure.

## Introduction

Compression of the circumflex artery by the sutures used for mitral valve repair is a well known, but a rare complication [1]. An early treatment should be the goal in treating the patients with iatrogenic circumflex artery injury following mitral valve (MV) repair in order to prevent the myocardial infarction [2]. Intraoperative transesophageal echocardiography (TEE) is a helpful diagnostic tool in making rapid diagnosis and management.

## Case Report

A 16-year-old woman with a past history of rheumatic heart disease was admitted to the hospital with progressive dyspnea on exertion. Preoperative transthoracic echocardiography showed severe mitral regurgitation, thickened anterior mitral leaflet, restricted mobility of posterior mitral leaflet and a normal left ventricular function. She was evaluated and scheduled for MV repair. After induction of anesthesia and endotracheal intubation, a TEE probe (Vivid S6, GE Medical Systems, Israel Ltd) was inserted. TEE examination confirmed the preoperative echocardiographic findings. MV repair was performed with augmentation of posterior mitral leaflet using glutaraldehyde treated pericardial patch and posterior mitral annuloplasty with 1 Bard^®^ Low Porosity, PTFE (polytetrafluoroethylene) Felt (IMPRA Inc, AZ, USA).

**Figure 1:**
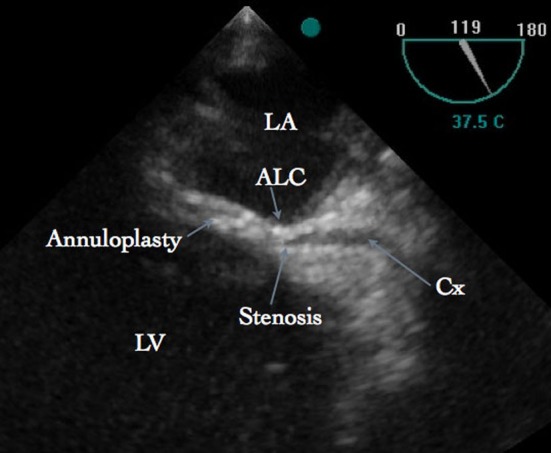
Transesophageal echocardiography showing stenosis of the circumflex artery. ALC, Anterolateral commissure; Cx, Circumflex artery; LA, Left Atrium; LV, Left Ventricle.

**Figure 2:**
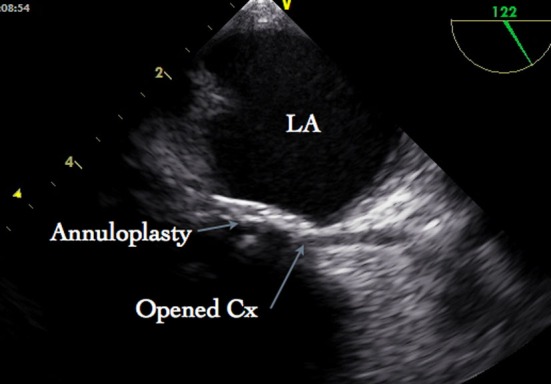
Transesophageal echocardiography after corrected procedure. Circumflex artery (Cx) is opened. LA, Left atrium.

After weaning from cardiopulmonary bypass (CPB), no mitral regurgitation or stenosis could be seen on TEE. However there was ST-segment elevation occurred in the Lead II, III and aVF. Further, TEE examination revealed dyskinesia of inferior, inferolateral and anterolateral segments of the left ventricle. A modified midesophageal long axis view was achieved with increasing the multiplane angle from 90 to 130 degrees and the TEE probe was slowly turned to the left of the patient to follow the course of the circumflex artery. In this view, the stenosis of the circumflex artery was observed near the anterolateral commissure (Fig. 1), which was not seen on the echocardiographic examination after induction of anesthesia and prior to CPB. Blood flow through the circumflex artery could not be demonstrated by color flow Doppler with decreasing the Nyquist limit to 40 cm/sec. Based on these findings, it was suggested that the first few sutures used for annuloplasty near the P1 region of the posterior annulus were responsible for narrowing of the vessel. Since the patient was hemodynamically unstable, an emergency coronary angiography could not be considered. An urgent CPB was re-instituted and the MV was re-explored. First two sutures near the P1 region were removed and translocated more superficially away from the annulus inwards the leaflets. Subsequently, the weaning from CPB was uneventful and the ST segment changes were normalized. The lumen of the circumflex artery near the anterolateral commissure is no longer compromised (Fig. 2) and regional wall motion abnormality was improved. Patient was extubated after 12 hours of operation and postoperative stay in the hospital was uneventful.

## Discussion

Iatrogenic injury of the circumflex artery is a recognized complication [1] and its incidence is ranging from 0.5 to 1.8% following mitral valve repair [2]. In MV surgery, the relationship between the posterior mitral valve annulus and the circumflex artery is extremely critical to prevent operative complications. In an earlier study, it was found that the shortest distance between the annulus and the coronary arteries was at the anterior commissure, where the circumflex artery was 3.9 ± 1.8 mm away from the annulus, while the longest distance (7.8 ± 2.6 mm) occurred at the posterior commissure of the mitral valve [3].

Various mechanisms have been proposed to cause injury of the circumflex artery during MV surgery. Injuries to the circumflex artery are caused by suture misplacement resulting in direct injury or distortion of the vessel during MV surgery as manifested in the present case. Other possible cause includes coronary embolization of air after open-heart surgery [4], which may be manifested as ST segment elevation in the electrocardiography and regional wall-motion abnormalities. However, these clinical manifestations are self-limiting and completely reversible with time.

**Figure 3:**
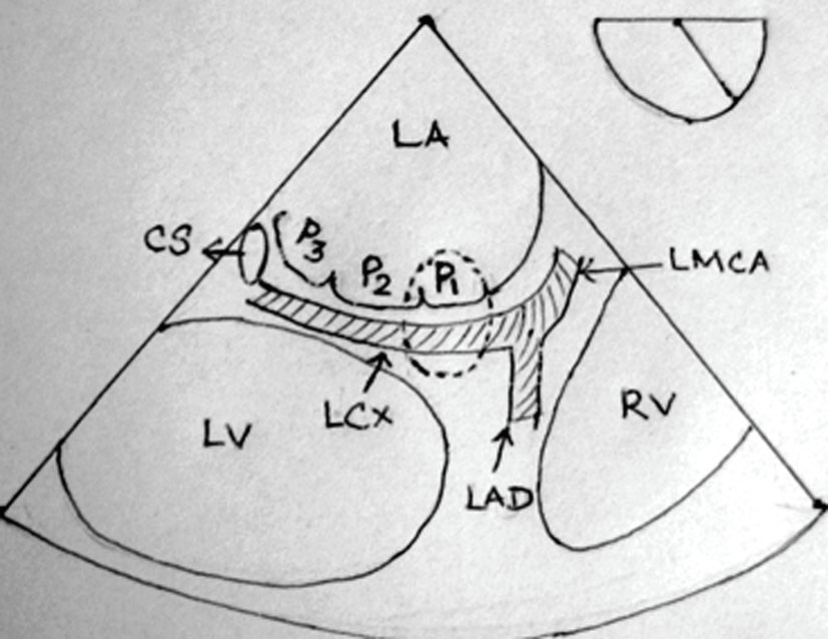
Schematic diagram of modified midesophageal long axis view shows commonest site (encircled) of circumflex artery injury. CS, Coronary sinus; LMCA, Left main coronary artery; LAD, Left anterior descending artery; LCx, Left Circumflex artery; LA, Left Atrium; LV, Left Ventricle; RV, Right Ventricle.

We report a case of compression of circumflex artery due to misplacement of sutures following MV repair and a rapid diagnosis was made based on the intraoperative TEE. With the use of modified midesophageal long axis view, the site of stenosis in the circumflex artery near the anterolateral commissure was demonstrated. As anterolateral commissure is being the commonest site of iatrogenic circumflex artery injury following MV repair, this view allows the adequate visualization of the circumflex artery (Fig. 3).

Previously, the circumflex artery injuries were diagnosed by performing an urgent coronary angiography and subsequently corrected procedures were performed [4, 5]. To the best of our knowledge, this is the first case report of circumflex artery injury following MV repair, which was diagnosed and intervened solely with the aid of TEE without performing the coronary angiography. The modified midesophageal long axis view along with electrocardiography provided early clue to this potentially fatal complication. When the diagnosis of the circumflex artery injury was possible, the decision to perform the corrected procedure could be made in the operating room without any delay. An early recognition of coronary compromise with TEE and surgical intervention rendered an uncomplicated postoperative course.
